# Essential oils and extracts of plants as biocides against microorganisms isolated from the ruins of the Roman city of Conímbriga (Portugal)

**DOI:** 10.1007/s11356-023-28212-6

**Published:** 2023-06-16

**Authors:** Dina M. R. Mateus, Eduardo Ferraz, Vera Perna, Pedro Sales, Virgílio Hipólito-Correia

**Affiliations:** 1 Technology, Restoration and Arts Enhancement Center (TECHN&ART), Instituto Politécnico de Tomar, Quinta Do Contador, Estrada da Serra, 2300-313 Tomar, Portugal; 2 Instituto Politécnico de Tomar, Quinta Do Contador, Estrada da Serra, 2300-313 Tomar, Portugal; 3 Museu Monográfico de Conímbriga, 3150-220 Condeixa-a-Velha, Portugal

**Keywords:** Biodeterioration, Built heritage, Color surface, Colorimetry, Essential oils, Green biocides, Mediterranean plants, Sustainable conservation and restoration

## Abstract

Biodeterioration of monumental complexes is in large part due to the proliferation of various microorganisms that attack the physical–chemical structures of support materials. Various conservation and restoration interventions use commercial biocides of synthetic origin, which exhibit some human and environmental toxicity and sometimes side effects on support materials. The main objective of this work is the assessment of new biocides obtained from endemic Mediterranean plants, to be used in the preservation of cultural heritage with the goal of contributing to the sustainable use of ecosystems and to the development of Mediterranean local communities. The biocidal potential of essential oils (EOs) and solvent extracts (SEs) (ethanol and n-hexane) obtained from four plants were evaluated: *Thymus mastichina* (Tm), *Mentha pulegium* (Mp), *Foeniculum vulgare* (Fv), and *Lavandula viridis* (Lv). Microorganisms collected at an emblematic site of Portuguese cultural heritage, the ruins of the Roman city of Conímbriga, were used to evaluate the biocidal activity of the EOs and SEs. It can be concluded that (i) SEs did not exhibit fungicidal nor bactericidal activity, except for one fungus specie; (ii) biocidal activity of EOs depends on the microorganism specie. The EOs showed a relative average biocidal activity (when compared to the commercial biocide Biotin T (1% v/v)) of 64%, 32%, 30%, and 25% for Mp, Fv, Lv, and Tm. On carbonate rocks, the application of Fv and Mp EOs up to 3 layers do not promote significant color/tonality changes in the surface of the rock. And the application of three layers of Lv and four layers of Fv, Mp, and Lv OEs only promote the occurrence of blurs or stains (variation of tonality) on rocks that presents very low porosity. It can also be noted that the EO of Mp has the broadest spectrum of activity. The results allow considering the use of Mp, Fv, Lv, and Tm EOs as valid alternatives to commercial biocides, providing a prospective application in the field of green conservation of building heritage.

## Introduction

The biodeterioration of cultural built heritage is in large part due to the proliferation of various microorganisms, often in symbiotic association, that excrete biotic compounds that attack the physical–chemical structures of support materials, such as organic acids (Warscheid and Braams 2000; Allsopp 2011; Sterflinger and Piñar 2013; Mateus et al. 2013; Meng et al. 2017; Municchia et al. 2018; Pina 2021).

This structural degradation is directly linked to climatic and environmental conditions, such as temperature, humidity, pollution, and solar exposition (Municchia et al. 2018, Pina 2021, Warscheid and Braams 2000). The Mediterranean climate is characterized by hot, dry summers, and mild wet winters (Lionello et al. 2006). The combination of high temperatures and high humidity in the summer months can create an ideal environment for the proliferation of microorganisms on outdoor surfaces, including the facades of built heritage (Pina 2021). Microorganisms, such as bacteria, fungi, and algae, can thrive on outdoor surfaces when they have access to moisture, nutrients, and a suitable temperature range. These microorganisms can cause deterioration of building materials and can also contribute to the formation of biofilms, which can affect the appearance and function of the buildings. In a protective and forestalling action, various conservation and restoration interventions aim to minimize this degradation of the support materials. In those interventions, different compounds of synthetic origin which exhibit some human and environmental toxicity are traditionally used, occasionally causing adverse effects on treated material (Warscheid and Braams 2000, Ashraf et al. 2014, Silva et al. 2016, Varnai et al. 2010, Fidanza and Caneva 2019).

Natural resource exploitation awareness and side effects of traditional commercial biocides led to the need for natural compounds with biocidal activity. Biocides with a wide spectrum of activity and low cost, favoring natural materials derived from plants which are generally considered easier to handle, more stable and less environmentally toxic, are currently preferred (Silva et al. 2016; Kakakhel et al. 2019).

**Fig. 1:**
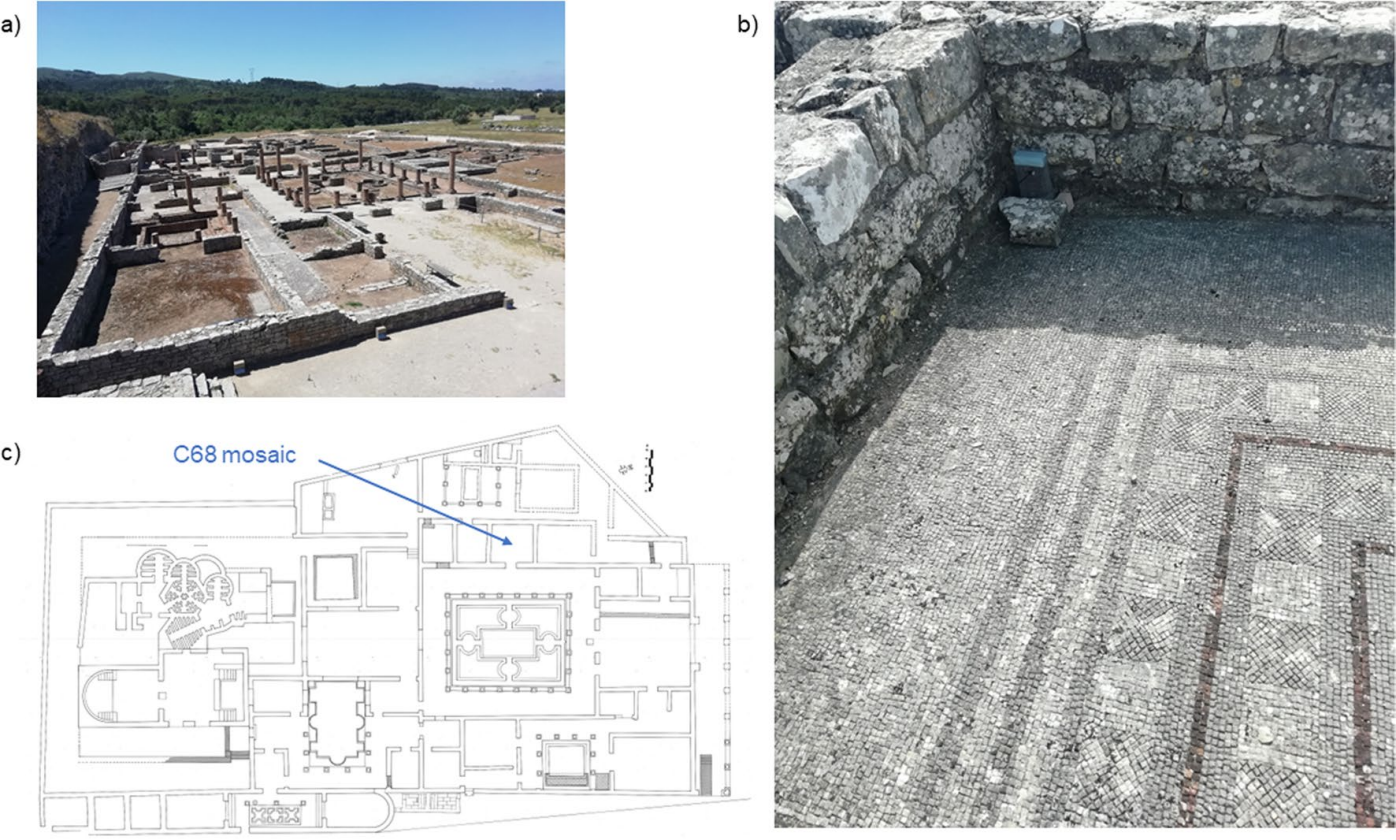
Photos of the House of Cantaber in the ruins of Roman city of Conímbriga (Portugal): **a** overview; **b** detail of the C68 mosaic; **c** plant

The main aim of the work is the evaluation and the application for new natural materials from Mediterranean plants that can be used as biocides in the preservation of cultural built heritage. Microorganisms collected from the ruins of the House attributed to Cantaber (HC) in the Roman city of Conímbriga were used to evaluate the biocidal activity. The HC (Fig. 1a) is the largest urban residence known in Conímbriga and is on par with the largest houses known in the west of the Roman Empire (3260 m^2^) (Correia 2020). The construction of this great residence took place in the Flavian period, in the last quarter of the I c. AD, occupying an insula that remained until then almost unoccupied, despite being centrally positioned in the urbanism of the Lusitanian city. The excavations of Conímbriga in 1930 began precisely in the area of the HC, and were followed by various restorations, carried out by the General Directorate of National Buildings and Monuments (Correia 2001). The mosaic floors of the HC have been, to a large extent, subject to lifting and consolidation in cement reinforced with iron bars. These actions took place between the end of the seventies and the beginning of the eighties of the last century. The C68 mosaic shows a composition with white, black, and red bands that delimit squares divided diagonally into alternating white and black triangles (Fig. 1b, c). All tesserae are made of limestone except for part of the red lines, which are made of ceramic. For the treatment of mosaics, quaternary ammonium-based biocides are periodically applied in stages to mitigate and/or eliminate the biofilms on their surface, followed by brushing and washing the tessellates with a neutral surfactant.

The application of untested substances on the surface of stones in cultural heritage always has a latent concern to the potential modification on the color/tonality of the stone surface. The colorimetry of the surface can be used to evaluate this potential color modification, as reported by Prieto et al. (2010) on granites, and by Fort et al. (2000) and Gabriele et al. (2021) on limestones from monuments.

A secondary goal of this work is related with the application of essential oils in modifying the color/tonality of the surface of carbonate rocks common and largely used in the build heritage presented on all the ruins of the Roman city of Conímbriga.

## Materials and methods

### Microorganism collection and characterization

The microorganisms used in the biocidal evaluation of the essential oils (EOs) and solvent extracts (SEs) were collected by swabbing (Verdier et al. 2014) from pigmented biofilms formed on the C68 mosaic of the HC (Correia 2001). The samples were collected from different locations selected by visual inspection of the pigmented areas. The swabs were immersed in 9 ml of sterile Ringer’s solution vortexed for 1 min and kept sealed at 4 °C for later usage.

Two media were used for isolation and subculture of heterotrophic microorganisms: Tryptic Soy Agar (TSA) (HiMedia) as a specific medium for bacteria; Potato Dextrose Agar (PDA) (HiMedia) supplemented with chlortetracycline for the growth of fungi. Autotrophic organisms were selected with BG-11 medium agar (Stanier and Cohen-Bazire 1997).

The microorganisms cultivable in the laboratory were identified by molecular biology techniques. Fungal colonies DNA extraction was performed with the REDExtract-N-Amp Plant PCR Kit (Sigma-Aldrich). ITS-rDNA region was amplified with the universal fungal primers ITS1-F/ITS4 (White et al. 1990; Gardes and Bruns 1993) and sequenced as a contracted service at STAB Vida (Caparica, Portugal). DNA sequences were processed with Geneious® R11.0.02 software and molecular similarity comparisons were obtained using the NCBI’s BLAST, with the option Standard nucleotide BLASTn of BLAST 2.6 (Altschul et al. 1997).

DNA extraction from bacterial colonies was performed with the DNeasy® Blood & Tissue Kit (Qiagen). The 16S rRNA gene was amplified with bacterial-specific primers 27F and 1525R (Rainey et al. 1996) and sequenced using the primers 519R, 803F, and 357F (Turner et al. 1999) as a contracted service at STAB Vida (Caparica, Portugal). The complete sequences were submitted to the NCBI’s BLAST with the option BLASTn of BLAST 2.6 for homology comparison (Altschul et al. 1997).

### Essential oils and solvent extract preparation

Biocidal activity of the EOs and SEs (ethanol and n-hexane) from the Portuguese endogenous plants *Thymus mastichina* (Tm), *Mentha pulegium* (Mp), *Foeniculum vulgare* (Fv), and *Lavandula viridis* (Lv) were evaluated.

The EOs were provided by the company Dalenguadiana, located in the Guadiana Valley Natural Park (Corte Sines, Mértola, Portugal). The EOs were produced in organically by hand-harvesting the plants and then by hydro-distilling in a Clavenger-type apparatus. The EOs composition was previously characterized through gas chromatography-mass spectrometry (Baptista et al. 2022). Other plant extracts, behind the essential oils, were obtained by solid–liquid extraction using the solvents ethanol and n-hexane and a Soxhlet-type apparatus, namely SEs.

### Assessment of antimicrobial activity

The evaluations of biocidal activity were performed by the agar disk-diffusion method. A volume of 200 μl of the microorganism’s suspension was smeared onto agar Petri dishes (10 cm diameter). Then, 0.6 cm filter paper disks (Macherey–Nagel) containing the tested EOs and SEs (20% v/v, emulsified with a solution of SDS 0.2%) were placed on the agar surface. The Petri dishes were incubated at 22 °C for 5 to 7 days, after which the diameters of growth inhibition zones were measured. The commercial biocide Biotin T (1% v/v) from CTS SRL (Altavilla Vicentina, Vicenza, Italia) was selected as positive control. A solution of DNS (0.2% m/v) in deionized water was used as a negative control for EOs assays. Aqueous solutions of DNS (0.2% m/v) and n-hexane or ethanol (20% v/v) were used as negative controls, for the trials with solvent extracts of ethanol and n-hexane, respectively.

### Evaluate of physical properties and potential color change of materials

Two types of carbonate rocks, a limestone from middle Jurassic (referred as MMC1) and a lacustrine calcareous tuff from Quaternary (referred as MMC2), both of local origin (Alarcão and Etienne 1977), were collected from the HC site. They were tested to evaluate physical properties and the potential color change with the addition of Fv, Mp, and Lv EOs. The rocks were cut on a saw disc to obtain four test samples with approximately 7 × 7 × 2 cm^3^ for the colorimetric analysis and six test samples with cubic shape (edge of 5 cm) for the physical analysis (water absorption at atmospheric pressure, open porosity, and apparent density). The surface of the probes was not subjected to any finishing. The samples for the colorimetric analysis were previously brushed, boiled in tapped water for two hours (to remove most of the organic matter) and dried at 100 ± 5 °C in a stove. The samples for the physical analysis were brushed with tapped water and dried at 70 ± 5 °C in a stove.

Colorimetric measurements were performed with a portable spectrometer Datacolor Check 3 (Lawrenceville, NJ, USA), with d/8º measuring geometry, 2 nm wavelength resolution, 10 nm effective bandwidth and spectral range from 360 to 700 nm (UV included). D65 CIE standard daylight illuminant (ISO 11664–2:^2007^), 10º CIE standard colorimetric observer (ISO/CIE 11664–1:^2019^), no gloss compensation, specular component excluded and small area view aperture size (10.0 mm illuminated–6.5 mm measured) were selected.

The laboratorial conditions for the colorimetric evaluation were temperature at 25 ± 5 °C and relative humidity at 28 ± 5%. The color tests were obtained by the average of five measurements at arbitrary locations on the surface of the samples (EN 15886:^2010^) and each measurement from the average of 3 flashes. The chromatic parameters were expressed in CIELAB (L*, a*, and b*) color space as defined on ISO/CIE 11664–4:^2019^ standard. For color analysis, it was calculated the total color differences ΔE*ab (or ΔE*76) according to EN 15886:2010. The chromatic parameters were evaluated before and after the EOs and Biotin T application on the surface of the test probes. The Biotin T was used as substance control for the comparison with the studied EOs. The surface of the probes was subdivided in four quadrants: the first was brushed with Biotin T; the second with Fv EO; the third with Mp EO; and the last with Lv EO. The EOs and Biotin T were applied once a week, during 4 weeks: one layer on the first week; two layers on the second week; three layers on the third week; and four layers on the fourth week. The increased number of layers on the surface of the test probe is equivalent to an increasing concentration of the EOs and Biotin T, after each application. The L*a*b* chromatic parameters express: L*, as lightness, with a lower limit of 0 (black) and an upper limit of 100 (white); a* is red (if a* > 0) or green (if a* < 0); and b* is blue (if b* < 0) or yellow (if b* > 0). ΔL*, Δa*, and Δb* calculate the difference of L*a*b* parameters before and after de application of EOs and Biotin T. And “delta E*ab” (ΔE*ab) quantify the total color difference before and after the application of EOs and Biotin T, according to the formula:

$${\Delta \mathrm{E}}^{*}\mathrm{ab}=\sqrt{{\left({\mathrm{L}}_{\mathrm{after}}^{*}-{\mathrm{L}}_{\mathrm{before}}^{*}\right)}^{2}+{\left({\mathrm{a}}_{\mathrm{after}}^{*}-{\mathrm{a}}_{\mathrm{before}}^{*}\right)}^{2}+{\left({\mathrm{b}}_{\mathrm{after}}^{*}-{\mathrm{b}}_{\mathrm{before}}^{*}\right)}^{2}}$$

Based on uniform color patches, some authors tried to correlate a ∆E*ab value to a Just Noticeable Difference (JND), to define a threshold detectable (perceived) by the human eye. Mahy et al. (1994) proposed a ∆E*ab = 2.3 to a JND, that we will take into consideration in the chromatic study.

The apparent density and open porosity were carried out according to NP EN 1936 (2008) and the water absorption at atmospheric pressure was accomplished to NP EN 13755 (2008). A threshold value of 10% for the coefficient of variation calculated for the set of probes was defined.

## Results and discussion

### Microorganism characterization

**Fig. 2:**
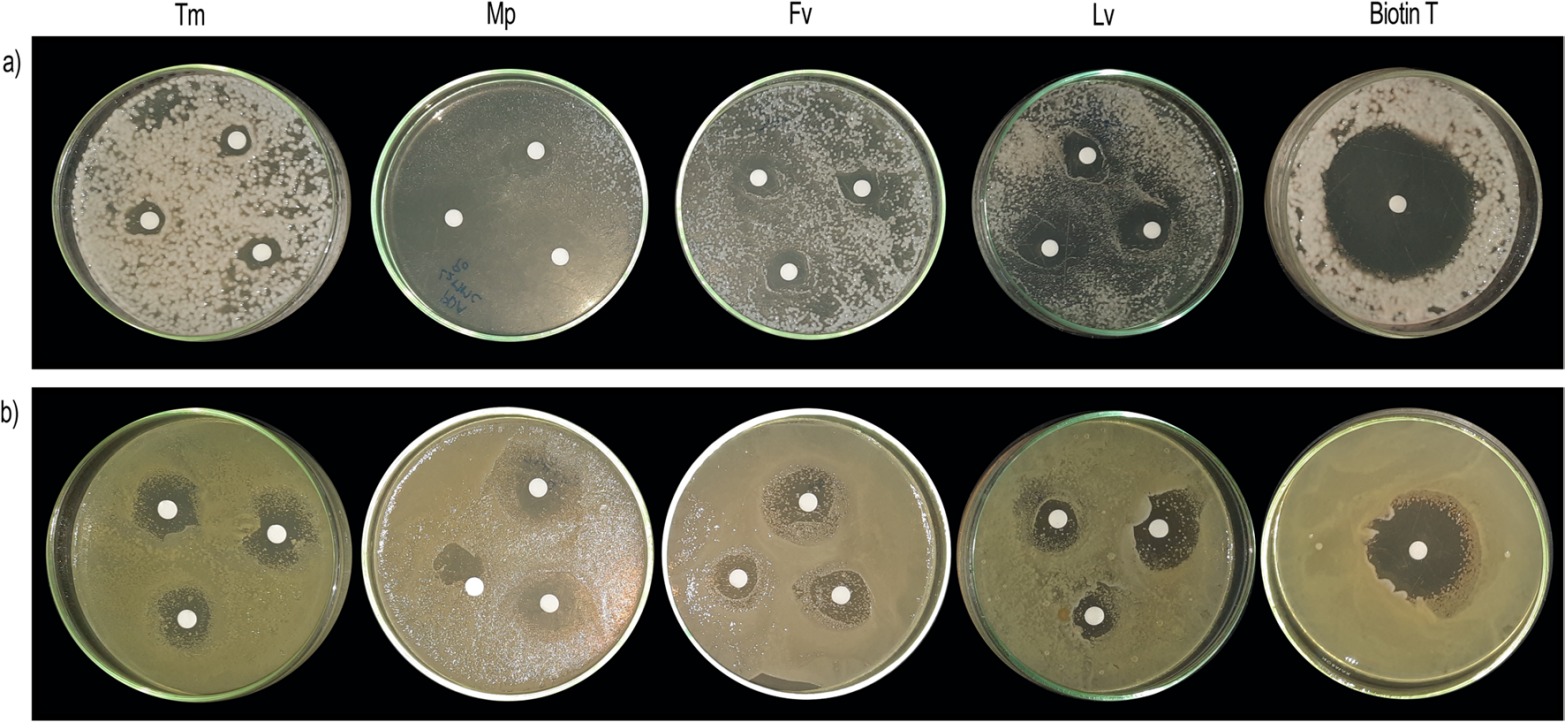
Inhibitory halo of growth for antimicrobial activity against **a** Y2 and **b** B2

A total of five filamentous fungi species (*Aspergillus versicolor*, F1; *Roussoella sp.*, F2; *Cladosporium cladosporioides*, F3; *Stagonosporopsis sp.*, F4; *Paraconiothyrium variabile*, F5), two yeasts species (*Cystobasidium minutum*, Y1; *Vishniacozyma globospora*, Y2) and two bacterial genera (*Pseudomonas,* B1; *Micobacterium*, B2) were isolated from the samples collected at the HC. No autotrophic microorganisms cultivable in the laboratory were detected. The samples collected showed a low level of microbial contamination, probably due to the periodic application of biocide as mentioned above. The results of the disk diffusion test are “qualitative,” in that a category of susceptibility is derived from the test (Palla et al. 2020; Balouiri et al. 2016). Thus, the diameter of the inhibition halos (IH) was used as a measure of the antimicrobial action (resistant (R) < 0.6 cm (diameter of Macherey–Nagel paper disks); 0.6 cm < sensible (S) < 10 cm; very sensible (VS) > 10 cm (diameter of Petri dish). Figure 2 shows examples of the growth-inhibition halos.

*Aspergillus versicolor*, *Roussoella* sp., *Cladosporium cladosporioides*, *Stagonosporopsis* sp., and *Paraconiothyrium variabile* are fungi that can be found on outdoor surfaces, including the facades of built heritage. *Aspergillus versicolor* had already been reported on limestone wall monuments in central Portugal (Trovão et al. 2019; Paiva et al. 2022), may play an important role in stone decay, and are usually associated with biodeterioration of cultural heritage (Pyzik et al. 2021). Bacteria belonging to the genera *Pseudomonas* and *Mycobacterium* were also recognized as biodeteriogenus of stone and wall paintings (Pyzik et al. 2021). *Cystobasidium minutum* is a species of fungi widespread in temperate regions of the world and is commonly found growing on decaying wood in moist habitats. *Vishniacozyma globospora* is a species of yeast found in soil, water, and decaying organic matter.

### Assessment of antimicrobial activity

**Fig. 3:**
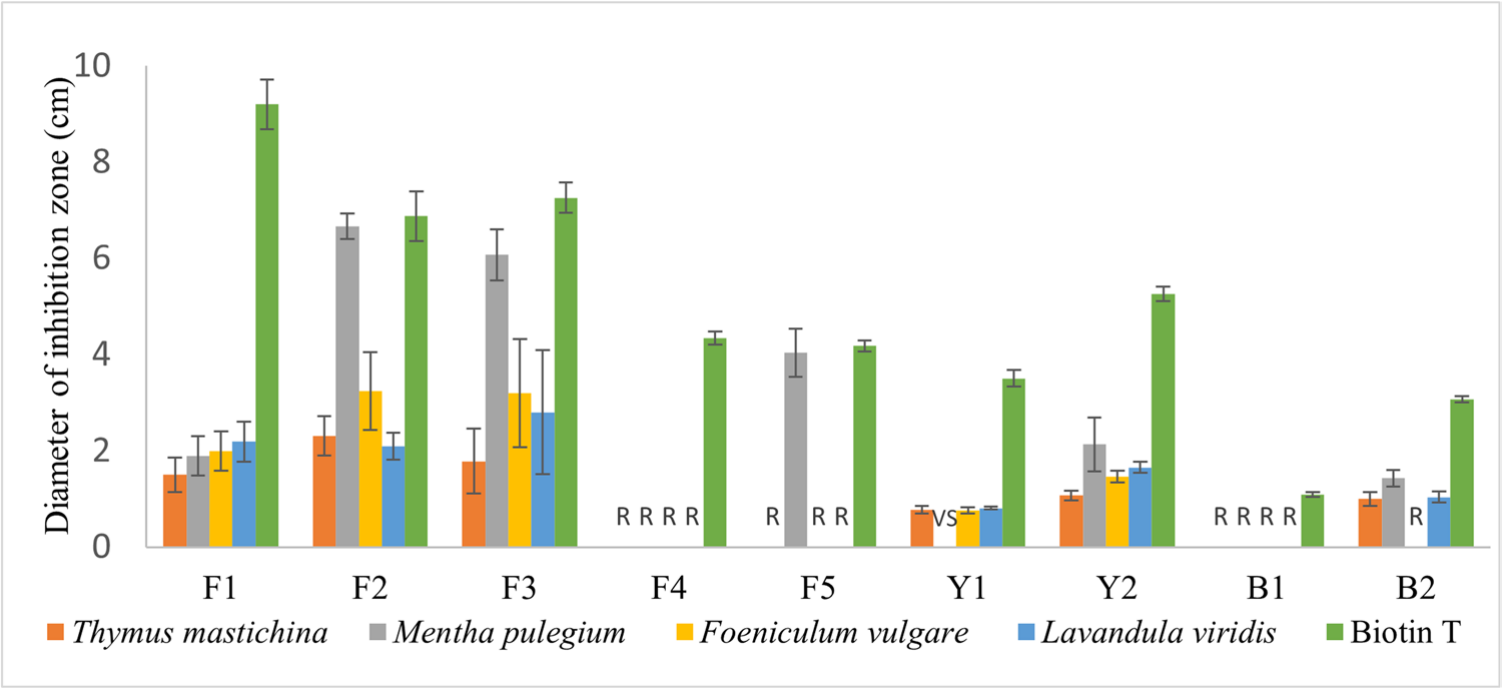
Median values of antimicrobial activity. Resistant (R) corresponds to inhibition halos < 0.6 cm and very sensible (VS) corresponds to inhibition halos > 10 cm

Table 1 and Fig. 3 show the results obtained for the antimicrobial activity of the evaluated EOs. The IH of the negative controls were lower than 0.6 cm (diameter of filter paper disks). SEs, n-hexane and ethanol, did not exhibit fungicidal nor bactericidal activity (IH < 0.6 cm), except for the fungi F2, which presents inhibition halos of 1.97 ± 0.37 cm, 1.90 ± 0.33 cm, and 1.29 ± 0.42 cm for the n-hexane extracts respectively of Tm, Fv, and Lv, and is resistant to Mp.

**Table 1:** Median values of antimicrobial activity of the EOs (20% v/v) emulsified in aqueous solution. (R) corresponds to inhibition halos < 0.6 cm (disks diameter) and very sensible (VS) corresponds to complete growth inhibition

Plant EOs	Diameter of inhibition zone (cm) ± 95% confidence interval											
	F1	F2	F3	F4	F5	Y1	Y2	B1	B2	F^(1)^	B^(2)^	All^(3)^
*Thymus mastichina*	1.5 ± 0.4	2.3 ± 0.4	1.8 ± 0.6	R	R	0.8 ± 0.1	1.1 ± 0.1	R	1.0 ± 0.1	0.23	0.32	0.25
*Mentha pulegium*	1.9 ± 0.4	6.7 ± 0.3	6.1 ± 0.5	R	4.0 ± 0.5	VS	2.1 ± 0.5	R	1.4 ± 0.2	0.68	0.46	0.64
*Foeniculum vulgare*	2.0 ± 0.4	3.2 ± 0.8	3.2 ± 1.1	R	R	0.8 ± 0.1	1.5 ± 0.1	R	R	0.32	R	0.32
*Lavandula viridis*	2.2 ± 0.4	2.1 ± 0.3	2.8 ± 1.2	R	R	0.8 ± 0.0	1.7 ± 0.1	R	1.0 ± 0.1	0.29	0.34	0.30
Biotin T (control)	9.2 ± 0.5	6.9 ± 0.5	7.3 ± 0.3	4.3 ± 0.3	4.2 ± 0.1	3.5 ± 0.2	5.3 ± 0.1	**1.1 ± 0.1**	3.1 ± 0.1	-	-	-

Relative average IH when compared to the Biotin T, excluding R and VS species: ^(1)^Fungus (including yeasts), ^(2)^bacteria, and ^(3)^all microorganisms

The biocidal activity of EOs depends on the plant type and microorganism species. The commercial biocide Biotin T was more effective than the EOs. This result is not unexpected due to the high toxicity of the active components of Biotin T (n-octyl-isothiazolinone and a quaternary ammonium salt). Natural biocides are intended to be a compromise that allows the control of the biodeterioration of cultural heritage while being more ecological and environmentally friendly.

Mp showed a biocidal activity against fungi F2, F3, and F5 close to Biotin T and higher to Biotin T against fungus F4. Mp EO proved to be the most effective, showing an inhibitory effect of 68% and 46% against fungi and bacteria, respectively. It can also be noted that the EO of Mp has the broadest spectrum of activity. In the literature, a low efficacy for *Menta piperita* against fungus of genera *Alternia*, *Aspergillus*, *Cladosporium*, *Penicillium*, and *Rhizopus* is reported (Levinskaitė and Paškevičius 2013). On the other hand, moderate antifungal activity was reported by Sakr et al. (2012) for *Mentha spicata* against fungi of genera *Candida*, *Saccharomyces*, and *Lodderomyces*.

The EOs did not show biocidal activity against F4 and B1 and only the Mp EOs showed biocidal activity against F5. Even Biotin T shows a low biocidal activity against B1.

Despite the importance of bacterial species in the biodeterioration of cultural heritage, very few studies address the use of EOs against bacterial species isolated from supporting materials of artworks and architectural heritage (Fidanza and Caneva 2019). The effectiveness of EOs reported in the literature is markedly variable, and this can be ascribed to the variability in the methodologies of testing (Fidanza and Caneva 2019; Palla et al. 2020; Veneranda et al. 2018).

### Evaluation of physical properties and surface color change of carbonate rocks

**Fig. 4:**
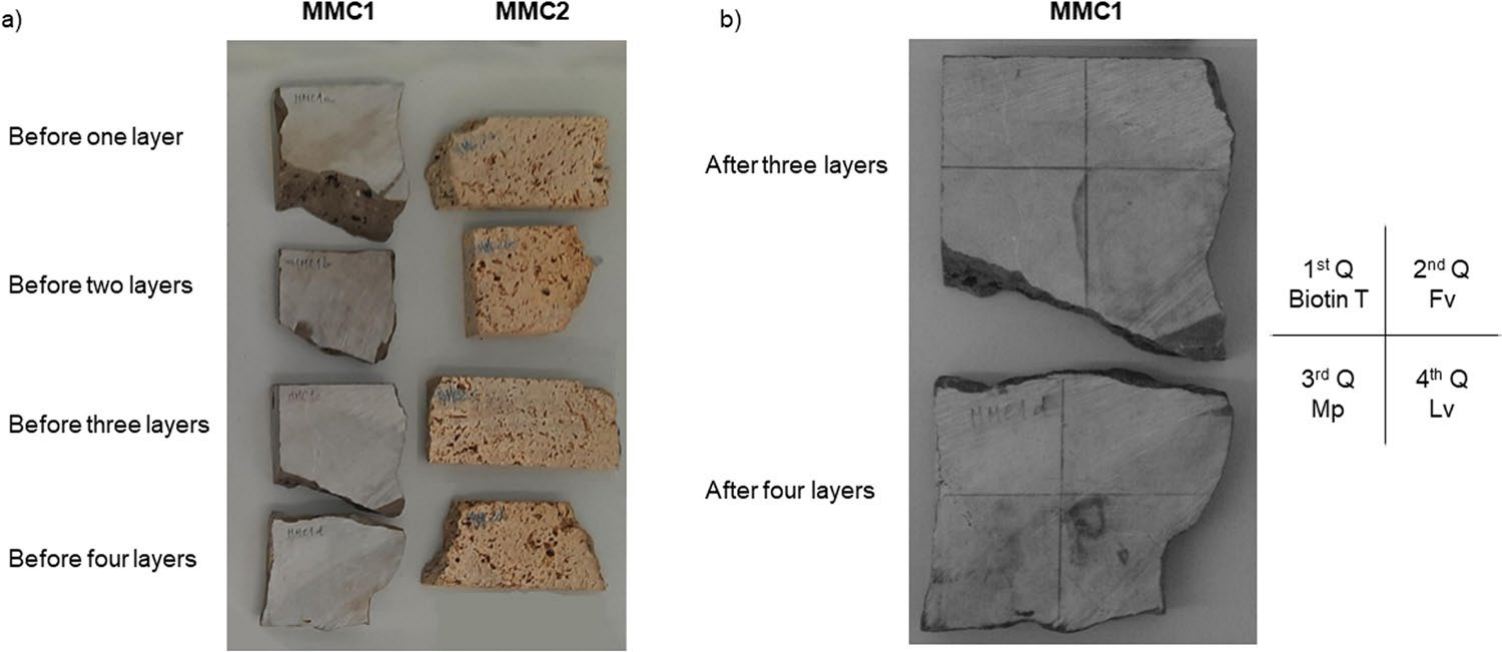
Chromatic test probes: **a** MMC1 and MMC2 samples before the application of EOs and Biotin T; **b** MMC1 sample after the application of three layers and four layers of EOs and Biotin T

Concerning the chromatic surface evaluation, MMC1 and MMC2 samples, and respective test probes, before the application of the EOs and Biotin T are shown in Fig. 4a. The impact of the three EOs that revealed the highest biocidal effect (*Foeniculum vulgare*, *Mentha pulegium*, and *Lavandula viridis*) was studied, as reported in previous section.

The application of Fv, Mp, and Lv EOs on the surface of MMC1 probes up to three layers (except for Lv EO), and on the surface of MMC2 probes up to four layers do not promote perceptible color variations. This fact is corroborated by the calculated ∆E*ab that presents values < 2.3 (Table 2). By other hand, the application of three layers of Lv EO and four layers of the EOs promote perceived changes on the color/tonality surface of the MMC1 probes (Fig. 4b). The application of Lv EO promotes the occurrence of a blur and a stain after the three (∆E*ab = 5.1) and four layers (∆E*ab = 9.4), respectively, evidenced by a pronounced decreasing of the lightness (∆L* =  − 4.8 and − 9.3, respectively). The same trend occurs (development of a stain) after applying four layers of Fv and Mp EOs (∆E*ab = 2.3 and 3.4, respectively), expressed also by a lightness decrease (∆L* =  − 2.2 and − 3.3, respectively).

**Table 2:** Chromatic parameter difference and respective total color difference for the carbonate rock samples

	One layer—1^st^ week				Two layers—2nd week				Three layers—3rd week				Four layers—4th week			
	∆L*	∆a*	∆b*	∆E*ab	∆L*	∆a*	∆b*	∆E*ab	∆L*	∆a*	∆b*	∆E*ab	∆L*	∆a*	∆b*	∆E*ab
MMC1																
Biotin T (control)	0.55	− 0.10	− 0.48	0.7	0.41	0.16	0.91	1.0	1.17	− 0.38	− 0.82	1.5	− 0.36	0.09	− 0.23	0.4
*Foeniculum vulgare*	− 0.95	0.18	− 0.10	1.0	− 2.04	− 0.21	− 0.22	2.1	− 1.29	0.02	0.03	1.3	− 2.23	− 0.27	− 0.26	2.3
*Mentha pulegium*	− 0.93	− 0.03	− 0.36	1.0	0.44	0.00	0.11	0.5	− 0.27	− 0.03	0.11	0.3	− 3.22	0.23	1.20	3.4
*Lavandula viridis*	− 0.43	0.07	0.12	0.4	0.82	− 0.25	− 0.57	1.0	− 4.83	0.63	1.51	5.1	− 9.25	0.84	1.27	9.4
MMC2																
Biotin T (control)	0.80	0.23	1.13	1.4	− 0.08	− 0.68	− 1.71	1.8	0.73	− 0.27	− 0.49	0.9	− 0.81	0.42	0.97	1.3
*Foeniculum vulgare*	0.10	0.13	0.55	0.6	1.70	− 0.44	− 0.81	1.9	− 0.66	− 0.26	− 0.55	0.9	1.24	− 0.03	− 0.08	1.2
*Mentha pulegium*	− 1.81	0.22	− 0.17	1.8	0.71	− 0.97	− 1.58	2.0	1.00	− 0.42	− 0.90	1.4	− 1.64	0.33	− 0.61	1.8
*Lavandula viridis*	0.18	− 0.49	− 0.28	0.6	− 1.24	0.06	− 0.14	1.2	1.37	0.09	1.00	1.7	− 1.15	0.31	0.68	1.4

As expected, the commercial and synthetic biocide Biotin T, during the colorimetric tests, do not promote any perceptible color/tonality change on the surface of the stone probes, and all the ∆E*ab values are under 2.3.

The apparent density, open porosity and water absorption at atmospheric pressure were evaluated on the MMC1 and MMC2 carbonate rocks (Table 3). When compared to MMC2, it is observed that MMC1 presents very low porosity (water absorption = 0.7% and open porosity = 1.5%) and consequently high compaction (apparent density = 2640 kg/m^3^). This result could influence the tonality variation promoted by the application of EOs, as previously described. In fact, the perception (and equipment evaluation) of blurs or stains (variations of tonality) is observed only in the sample with very lower porosity, probably due to the “sorption” of the non-volatile components of the EOs on the surface of the rock. Rocks with high porosity, as MMC2 sample, tend to dissimulate this alleged “sorption” inside the pores, and in this case, the changes in color/tonality are not perceptible by human eye and by the used test equipment. Further studies should be performed to confirm this preliminary interpretation.

**Table 3:** Physical properties of the carbonate rock samples

Rock sample	Water absorption at atmospheric pressure (%)	Open porosity (%)	Apparent density (kg/m^3^)
MMC1	0.7 ± 0.1	1.5 ± 01	2640 ± 20
MMC2	9.1 ± 0.8	16.3 ± 16*	1960 ± 110*

*Due to macroscopic porosity, the apparent volume was calculated by cubature (according to NP EN 1936:^2008^)

## Conclusions

For the microorganisms isolated from the samples collected on the floor mosaic ruins of the CH and for the tested conditions (20% of EOs, n-hexane or ethanol plant extracts, emulsified with a solution of SDS 0.2%), it can be concluded that ethanol extracts for the four tested plants do not exhibit fungicidal nor bactericidal activity and n-hexane extracts only exhibit microbial activity against the fungi F2. Biocidal activity of EOs depends on the plant type and microorganism species.

The EOs showed a relative average biocidal activity, when compared to the commercial biocide Biotin T, of 64%, 32%, 30%, and 25% respectively for Mp, Fv, Lv, and Tm and for all microorganisms assayed; 68%, 32%, 29%, and 23% respectively for Mp, Fv, Lv, and Tm, considering all fungi assayed; 46%, 34%, and 32% for Mp, Lv, and Tm, respectively, considering the two bacteria assayed. It can also be noted that the EO of Mp has the highest and broadest spectrum of activity. The results allow considering the use of Mp, Fv, Lv, and Tm EOs as valid alternatives to commercial biocides, providing a prospective application in the field of green conservation of building heritage. Moreover, the use of these plants to produce natural biocides may provide a unique opportunity for local development and contributes to the sustainable use of ecosystems, fighting desertification in the Mediterranean regions.

In general, with carbonate rocks, the application of Fv and Mp EOs in a lower quantity (up to 3 layers) do not promote significant color/tonality changes in the surface of the rock. But the application of three layers of Lv and four layers (higher quantity) of Fv, Mp, and Lv OEs promotes the occurrence of blurs or stains in the surface of a carbonate rocks with very lower porosity.

The results allow considering the use of Mp, Fv, Lv, and Tm EOs as valid alternatives to commercial biocides, providing a prospective application in the field of green conservation of building heritage. Moreover, the use of these plants to produce natural biocides may provide a unique opportunity for local development and contributes to the sustainable use of ecosystems, fighting desertification in the Mediterranean regions.

## Data Availability

The datasets used and/or analyzed during the current study are available from the corresponding author on reasonable request.

## References

[CR1] Fouilles de Conimbriga I: L'architecture. Paris, De Boccard

[CR2] Allsopp D (2011). Worldwide wastage: the economics of biodeterioration. Microbiol Tod.

[CR3] Altschul SF, Madden TL, Schäffer AA, Zhang J, Zhang Z, Miller W, Lipman DJ (1997). Gapped BLAST and PSI-BLAST: a new generation of protein database search programs. Nucleic Acids Res.

[CR4] Ashraf MA, Ullah S, Ahmad I, Qureshi AK, Balkhair KS, Rehman MA (2014). Green biocides, a promising technology: current and future applications to industry and industrial processes. J Sci Food Agr.

[CR5] Balouiri M, Sadiki M, Ibnsouda SK (2016). Methods for in vitro evaluating antimicrobial activity: A review. J Pharmaceut Anal.

[CR6] Baptista C, Santos L, Amaral ME, Silva L (2022) Chemical characterization of essential oils with a biocide base for conservation and restoration. In: 1st International FibEnTech Congress (FibEnTech21) New opportunities for fibrous materials in the ecological transition, KnE Materials Science, pp 80–90. 10.18502/kms.v7i1.11611

[CR7] Correia VH (2001). Conímbriga, Casa atribuída a Cantaber. Trabalhos arqueológicos 1995–1998. Conimbriga.

[CR8] Correia VH (2020) Conímbriga (Condeixa-a-Velha, Portugal). In: Pizzo A (ed) La arquitectura doméstica urbana de la Lusitania Romana, Mytra 6, Mérida, pp 273–295.

[CR9] EN 15886:2010 Conservation of cultural property - test methods – color measurement of surfaces. European Committee for Standardization, Brussels.

[CR10] Fidanza M, Caneva G (2019). Natural biocides for the conservation of stone cultural heritage: a review. J Cult Herit.

[CR11] Fort RA, Mingarro F, López de Azcona MC, Rodriguez Blanco J (2000). Chromatic parameters as performance indicators for stone cleaning techniques. Color Res Appl.

[CR12] Gabriele F, Tortora M, Bruno L, Casieri C, Chiarini M, Germani R, Spreti N (2021). Alginate-biocide hydrogel for the removal of biofilms from calcareous stone artworks. J Cult Heritage.

[CR13] Gardes M, Bruns TD (1993). ITS primers with enhanced specificity for basidiomycetes application to the identification of mycorrhizae and rusts. Mol Ecol.

[CR14] ISO 11664–2:2007 Colorimetry - Part 2: CIE standard illuminants**.** International Organization for Standardization, Switzerland.

[CR15] ISO/CIE 11664–1:2019 Colorimetry - Part 1: CIE standard colorimetric observers. International Organization for Standardization, Switzerland.

[CR16] ISO/CIE 11664–4:2019 Colorimetry - Part 4: CIE 1976 L*a*b* colour space. International Organization for Standardization, Switzerland.

[CR17] Kakakhel M, Wu F, Gu J, Feng H, Shah K, Wang W (2019) Controlling biodeterioration of cultural heritage objects with biocides: a review. Int Biodeterior Biodegrad 143:104721. 10.1016/j.ibiod.2019.104721

[CR18] Levinskaitė L, Paškevičius A (2013). Fungi in water-damaged buildings of Vilnius Old City and their susceptibility towards disinfectants and essential oils. Indoor Built Environ.

[CR19] Lionello P, Malanotte-Rizzoli P, Boscolo R, Alpert P, Artale V, Li L, Luter-Bacher J, May W, Trigo R, Tsimplis M, Ulbrich U, Xoplaki E (2006) The Mediterranean climate: an overview of the main characteristics and issues. In: Lionello P, Malanotte-Rizzoli P, Boscolo R (ed) Developments in Earth and Environmental Sciences 4, Elsevier, Amsterdam, pp 1-26

[CR20] Mahy M, Van Eycken L, Oosterlinck A (1994). Evaluation of uniform color spaces developed after the adoption of CIELAB and CIELUV. Color Res Appl.

[CR21] Mateus DMR, Silva RB, Costa FMC, Coroado JPF (2013) Microbiological diversity in the Unfinished Sacristy building of the Convent of Christ, Tomar, and evaluation of its biocide-based control. Cons Património 17:11–20. 10.14568/cp2012005 (in portuguese)

[CR22] Meng H, Katayama Y, Gu JD (2017). More wide occurrence and dominance of ammonia-oxidizing archaea than bacteria at three Angkor sandstone temples of Bayon, Phnom Krom and Wat Athvea in Cambodia. Int Biodeter Biodegr.

[CR23] Municchia AC, Bartoli F, Taniguchi Y, Giordani P, Caneva G (2018). Evaluation of the biodeterioration activity of lichens in the cave Church of Üzümlü (Cappadocia, Turkey). Int Biodeter Biodegr.

[CR24] NP EN 1936 (2008) - Natural stone test methods. Determination of real density and apparent density, and of total and open porosity. Instituto Português da Qualidade, Caparica, Portugal.

[CR25] Natural stone test methods. Determination of water absorption at atmospheric pressure. Instituto Português da Qualidade, Caparica, Portugal

[CR26] Paiva DS, Fernandes L, Trovão J, Mesquita N, Tiago I, Portugal A (2022). Uncovering the Fungal Diversity Colonizing Limestone Walls of a Forgotten Monument in the Central Region of Portugal by High-Throughput Sequencing and Culture-Based Methods. Appl Sci.

[CR27] Palla F, Bruno M, Mercurio F, Tantillo A, Rotolo V (2020). Essential oils as natural biocides in conservation of cultural heritage. Molecules.

[CR28] Pina D (2021) Microbial growth and its effects on inorganic heritage materials. In: Joseph E (ed) Microorganisms in the deterioration and preservation of cultural heritage, 1st edn, Springer, Neuchâtel, pp 3-35

[CR29] Prieto B, Sanmartín P, Silva B, Martinez-Verdú F (2010). Measuring the color of granite rocks: a proposed procedure. Color Res Appl.

[CR30] Pyzik A, Ciuchcinski K, Dziurzynski M, Dziewit L (2021). The bad and the good - microorganisms in cultural heritage environments - an update on biodeterioration and biotreatment approaches. Mater.

[CR31] Rainey F, Ward-rainey N, Kroppenstedt R (1996). The Genus *Nocardiopsis* represents a phylogenetically coherent taxon and a distinct actinomycete lineage: ProDosal of *Nocardiomaceae* fam. nov. Int J Syst Bacteriol.

[CR32] Sakr AA, Ghaly M, Abdel-Haliem M (2012). The efficacy of specific essential oils on yeasts isolated from the Royal Tomb paintings at Tanis Egypt. Int J Conserv Sci.

[CR33] Silva M, Pereira A, Teixeira D, Candeias A, Caldeira AT (2016). Combined use of NMR, LC-ESI-MS and antifungal tests for rapid detection of bioactive lipopeptides produced by Bacillus. Adv Microbiol.

[CR34] Stanier R, Cohen-Bazire G (1997). Phototrophic prokaryotes: the cyanobacteria. Ann Rev Microbiol.

[CR35] Sterflinger K, Piñar G (2013). Microbial deterioration of cultural heritage and works of art - tilting at windmills?. Appl Microbiol Biotechnol.

[CR36] Trovão J, Portugal A, Soares F, Paiva DS, Mesquita N, Coelho C, Pinheiro AC, Catarino L, Gil F, Tiago I (2019). Fungal diversity and distribution across distinct biodeterioration phenomena in limestone walls of the old cathedral of Coimbra, UNESCO World Heritage Site. Int Biodeter Biodegr.

[CR37] Turner S, Pryer KM, Miao VP, Palmer JD (1999). Investigating deep phylogenetic relationships among cyanobacteria and plastids by small subunit rRNA sequence analysis. J Eukaryotic Microbiol.

[CR38] Varnai VM, Macan J, Ljubičić Ćalušić A, Prester L, Kanceljak Macan B (2010). Upper respiratory impairment in restorers of cultural heritage. Occup Med.

[CR39] Veneranda M, Blanco-Zubiaguirre L, Roselli G, Di Girolami G, Castro K, Madariaga JM (2018). Evaluating the exploitability of several essential oils constituents as a novel biological treatment against cultural heritage biocolonization. Microchem J.

[CR40] Verdier T, Coutand M, Bertron A, Roques C (2014). A review of indoor microbial growth across building materials and sampling and analysis methods. Build Environ.

[CR41] Warscheid Th, Braams J (2000). Biodeterioration of stone: a review. Int Biodeter Biodegr.

[CR42] White T, Bruns T, Lee S, Taylor J (1990) Amplification and direct sequencing of fungal ribosomal RNA genes for phylogenetics. In: Gelfand D, Shinsky J, White T (ed), PCR Protocols: A Guide to Methods and Applications, Academic Press, London, pp 315-322

